# The Fuzzy Kinetics Index: an indicator conflating cardiorespiratory kinetics during dynamic exercise

**DOI:** 10.1007/s00421-021-04611-w

**Published:** 2021-02-18

**Authors:** U. Drescher

**Affiliations:** German Sport University Cologne, Am Sportpark Müngersdorf 6, 50933 Cologne, Germany

**Keywords:** Circulation, Pseudo random binary sequence (PRBS), Gas exchange, Time series analysis, Exercise tolerance, Sport performance

## Abstract

**Purpose:**

The aim of the present study was to develop a novel index using fuzzy logic procedures conflating cardiorespiratory and pulmonary kinetics during dynamic exercise as a representative indicator for exercise tolerance.

**Methods:**

Overall 69 data sets were re-analyzed: (age: 29 ± 1.2 y [mean ± SEM], height: 179 ± 1.0 cm; body mass: 78 ± 1.4 kg; peak oxygen uptake ($\dot {\text{V}}$˙O_2peak_): 48 ± 1.1 ml·min^−1^·kg^−1^), that comprised pseudo random binary sequence work rate (WR) changes between 30 and 80 W on a cycle ergometer, with additional voluntary exhaustion to estimate $\dot {\text{V}}$O_2peak_. Heart rate (HR), stroke volume (SV) and gas exchange (pulmonary oxygen uptake ($\dot {\text{V}}$O_2pulm_)) were measured beat-to-beat and breath-by-breath, respectively. For estimation of muscle oxygen uptake ($\dot {\text{V}}$O_2musc_) kinetics and for the analysis of kinetic responses of the parameters of interest (perfusion ($\dot {\text{Q}}$ = HR·SV), $\dot {\text{V}}$O_2pulm_, $\dot {\text{V}}$O_2musc_) the approach of Hoffmann et al. (2013) was applied. For calculation of the Fuzzy Kinetics Index $\dot {\text{Q}}$, $\dot {\text{V}}$O_2pulm_, and $\dot {\text{V}}$O_2musc_ were used as input variables for the subsequent fuzzy- and defuzzyfication procedures.

**Results:**

For both absolute and relative $\dot {\text{V}}$O_2peak_ a significant correlation has been observed with FKI, whereas the correlation coefficient is higher for relative (r = 0.430; p < 0.001; n = 69) compared to absolute $\dot {\text{V}}$O_2peak_ (r = 0.358; p < 0.01; n = 69). No significant correlations have been found between FKI and age, height or body mass (p > 0.05 each).

**Conclusions:**

The significant correlations between FKI and $\dot {\text{V}}$O_2peak_ represent a physiological connection between the regulatory and the capacitive system and its exercise performance. In turn, the application of FKI can serve as an indicator for healthy participants to assess exercise tolerance and sport performance.

**Supplementary Information:**

The online version contains supplementary material available at 10.1007/s00421-021-04611-w.

## Introduction

Oxygen uptake ($\dot {\text{V}}$O_2_) is an essential parameter for the capacitive variables (e.g., maximal ($\dot {\text{V}}$O_2max_) or peak ($\dot {\text{V}}$O_2peak_) oxygen uptake) and the regulation characteristics (e.g., kinetic responses) of the cardiorespiratory and pulmonary system (Jones & Poole 2013). Therefore, these parameters can be used as gross proxy of the individuals’ exercise tolerance and sport performance (Wasserman 1984; Bassett & Howley 2000).

However, $\dot {\text{V}}$O_2max_ or $\dot {\text{V}}$O_2peak_ represents the combined performance of the muscle, circulatory and other O_2_-consuming systems in a single value. These values are, therefore, the gross criterion of the cardiorespiratory and pulmonary systems as indicator of the aerobic metabolism. In this regard, $\dot {\text{V}}$O_2max_ or $\dot {\text{V}}$O_2peak_ cannot be subdivided into its underlying physiological systems.

In contrast, the kinetics analysis introduced by Hoffmann et al. (2013) allows to distinguish between cardiac output ($\dot {\text{Q}}$), pulmonary ($\dot {\text{V}}$O_2pulm_), and muscle ($\dot {\text{V}}$O_2musc_) oxygen uptake kinetics. These components represent, therefore, the respective shares of the cardiorespiratory and pulmonary systems.

To combine these three kinetic parameters expressed as one representative physiological index, fuzzy logic basics can be applied (Zadeh 1965). The proceedings of fuzzy logic can be applied due to the regulative characteristics of kinetic responses and in particular the fuzzy ─ non-linear ─ distortions between $\dot {\text{V}}$O_2musc_ and $\dot {\text{V}}$O_2pulm_ kinetics (Benson et al. 2013, 2017).

Therefore, the aim of the present study was to develop a novel index using fuzzy logic procedures conflating cardiorespiratory and pulmonary kinetics during dynamic exercise as representative indicator for exercise tolerance and sport performance.

Further, the presented Fuzzy Kinetics Index (FKI) will be applied to real physiological data to establish correlations with $\dot {\text{V}}$O_2peak_ and additional parameters of interest (e.g., age, height, body mass, body mass index).

The following hypotheses will be tested:

- A significant moderate correlation will be demonstrated between absolute $\dot {\text{V}}$O_2peak_ and the proposed FKI.
- A significant high correlation will be demonstrated between relative $\dot {\text{V}}$O_2peak_ and FKI.
- No significant correlations will be observed between FKI and age, height, body mass or body mass index.

In addition, a classification scheme will be proposed to subdivide the FKI as comprised individual kinetic responses into five sections (very slow, slow, medium, fast, and very fast).

## Methods

### Study participants

For calculation of FKI, data of previous publications have been applied and re-analyzed. In total, six publications with an overall sample size of 69 participants (see Table 1) are involved in the present study (Drescher et al. 2015, 2016, 2017, 2018a, 2018b, 2018c).

**Table 1:** Participants’ characteristics (n = 69)

Parameter	Mean ± SD
Age [y]	29 ± 10
Height [cm]	179 ± 8
Body mass [kg]	78 ± 12
Body mass index [kg·m^−2^]	24 ± 3
$\dot {\text{V}}$O_2peak_ [L·min^−1^]	3.7 ± 0.8
$\dot {\text{V}}$O_2peak_ [ml·min^−1^·kg^−1^]	48 ± 9

The studies were approved by the Ethics Committee of the German Sport University Cologne and all procedures were in accordance with the 1964 Helsinki Declaration and its later amendments or comparable ethical standards. All subjects gave their written informed consent prior to participating in the exercise test.

### Dynamic exercise protocol

**Fig. 1:**
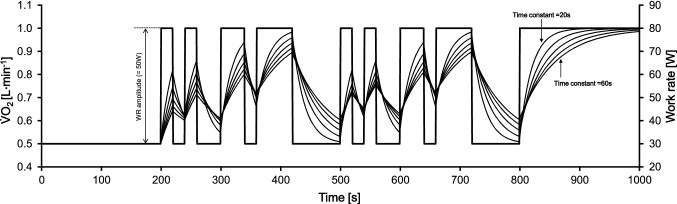
Dynamic exercise protocol with two pseudo-random binary sequences (PRBS, thick line), implying work rate (WR) changes between 30 and 80 W, represented as a WR amplitude of 50 W. Additionally, five different idealized kinetic responses of oxygen uptake ($\dot {\text{V}}$O_2_, small lines) implying time constants from 20 to 60 s (step width: 10 s) without delay time are displayed. Time constants reflect the time to attain 63% of the WR amplitude

The exercise protocol of all involved studies comprised a series of two pseudo-random binary sequence (PRBS) work rate (WR) changes between 30 and 80 W (see Fig. 1 for exercise protocol). Before and after the PRBS, constant WR phases at 30 and 80 W (200 s each) were defined for steady state estimations, which are not of relevance for the current FKI analysis and will not be considered further.

$\dot {\text{V}}$O_2peak_ was estimated by subsequent step increases in WR following the PRBS WR changes. In 11 of 69 cases the step increases were performed by 50 W · 5 min^−1^ and in the other remaining 58 cases by 25 W · min^−1^ until the subjects reached their symptom limited maximum.

All tests were performed on bicycle ergometers (Cardiac Stress Table, Lode B.V., Netherlands; Ergometrics er900L, Schiller AG, Baar, Switzerland; ErgoFit 407, ERGO-FIT GmbH & Co. KG, Pirmasens, Germany) with a backrest at 45° and a leg ergometer device at 42° to the ground. With regard to various body positions in one study (Drescher et al. 2016), the leg ergometer device was mounted firmly on the tilt table so that it maintained an angle of 45° to the ground when the body position was at the same angle.

In all studies the cadence was set to 60 rpm during the PRBS phases.

### $\dot {{V}}$*O*_*2pulm*_*, heart rate and stroke volume measurements*

$\dot {\text{V}}$O_2pulm_ was measured by breath-by-breath gas exchange (ZAN 680, ZAN Messgeräte GmbH, Oberthulba, Germany) and heart rate (HR) by beat-to-beat electrocardiogram (ECG: R-R intervals; TaskForce® Monitor, CNSystems Medizintechnik AG, Austria; and Schiller AT-104 PC EKG, Schiller Medizintechnik GmbH, Feldkirchen, Germany). Stroke volume (SV) was assessed by different approaches (Fortin et al. 2006; Lentner 1990; Rühle et al. 1983; Whipp et al. 1996). $\dot {\text{Q}}$ was then calculated by multiplying HR with SV.

### *Time series analysis and estimation of *$\dot {{V}}$*O*_*2musc*_* kinetics*

Commonly, $\dot {\text{V}}$O_2_ kinetic responses are estimated by using step increases in WR and following analysis of time constants (τ), reflecting the time to attain 63% of the WR amplitude.

Alternatively, the kinetic responses of physiologic parameters can be calculated as introduced by Hoffmann et al. (2013). In doing so, the procedures of time series analysis (auto- (ACF), cross-correlation function (CCF)), and the PRBS WR protocol are applied in combination with a circulatory transfer model to estimate $\dot {\text{V}}$O_2musc_ by means of $\dot {\text{Q}}$ and $\dot {\text{V}}$O_2pulm_ measurements. This approach was needed due to the fact that $\dot {\text{V}}$O_2pulm_ kinetics are non-linearly time-delayed and distorted compared to $\dot {\text{V}}$O_2musc_ kinetics, which is based on transient venous return and $\dot {\text{Q}}$ dynamics (Lador et al. 2006; Hoffmann et al. 2013; Drescher 2018).

The ACF of WR was estimated using the two PRBS WR phases. Precisely, for all 300 shifts (lags), the correlation coefficients have been calculated according to the time series analysis definition. In doing so, the resulting ACF of WR describes a triangular shape, which is interpreted as increasing and decreasing WR protocol in the correlation domain.

**Fig. 2:**
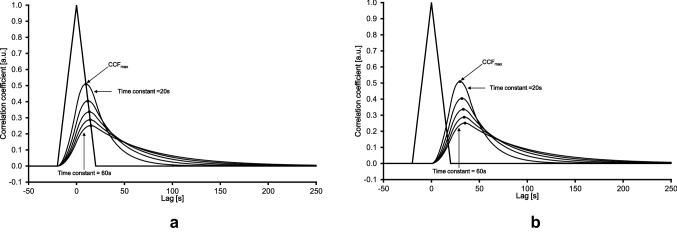
Auto-correlation function (ACF, triangular shape) of work rate (WR) and cross-correlation functions (CCF, curvaceous shapes) between WR and five different idealized kinetic responses implying time constants from 20 to 60 s (step width: 10 s) from Fig. 1. The CCF responses are displayed without (2A) and with (2B) delay time; CCF_max_: maximum of CCF (black dots)

Accordingly, the CCF of the measured physiologic parameters ($\dot {\text{Q}}$, $\dot {\text{V}}$O_2pulm_, $\dot {\text{V}}$O_2musc_) were correlated with the PRBS WR protocol in each case. Correspondingly, the CCF resulted in an increasing and decreasing course, with an absolute maximum (CCF_max_), which indicates the speed of the kinetic response of the respective parameter. In this regard, higher CCF_max_ values represent faster and smaller CCF_max_ values represent slower kinetic responses. In the present work, the CCF_max_ values are used as proxy for the speed of the kinetic responses of the respective physiologic parameter. In principle, the CCF is interpreted as the response to the ACF. Figure 2 illustrates five different kinetic responses with τ ranging from 20 to 60 s (step width: 10 s), both without (Fig. 2A) and with (Fig. 2B) delay times. Noteworthy, the pure delay time has no impact on the CCF_max_ value and indicates a simple right-shift of the CCF course.

Further, the ACF is an approximation to the Dirac impulse, used to identify the underlying system characteristics. In this way, a series of PRBS can be applied to analyze the features of the physiologic systems involved during dynamic exercise.

For comparison between the traditional (step responses) and the PRBS (time-series) approach, the CCF_max_ values were transformed into τ values. The following complex equation was used for this purpose, based on the publication by Drescher (2012):

1 $$ {\text{CCF}}_{{\max }}  = 2 - \frac{1}{{20}}\left[ {\tau  \cdot \log \left( { - 1 + 2e^{{\frac{{20}}{\tau }}} } \right)} \right] $$

where CCF_max_ is the peak of the cross-correlation function, *τ* is time constant, *log* is natural logarithm, *e* is Euler’s number, and the number *20* represents the shortest element of the PRBS WR protocol, which is 20 s. To calculate τ, a concrete value for CCF_max_ must be used, and then the equation must be solved for τ.

### Calculation of Fuzzy Kinetics Index (FKI)

**Fig. 3:**
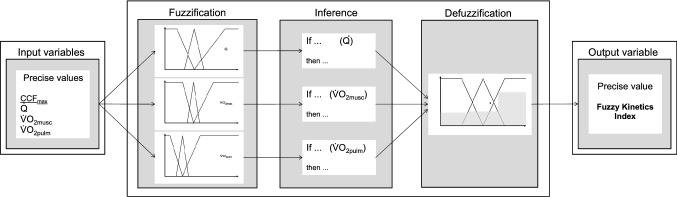
Process flow to calculate the Fuzzy Kinetics Index (FKI). For detailed explanation see text

To calculate FKI a fuzzy control process flow was used as displayed in Fig. 3. In the first step (fuzzification) input variables are needed to determine the fuzzy sets. The second step (inference) implies pre-defined calculation rules to determine the degree of affiliation of a property (fast to slow kinetics).

The third step (defuzzification) comprises the conversion of fuzzy quantities into concrete numerical values, which results in the output variable FKI. The different steps are described in detail below.

All FKI calculations have been performed with Microsoft Excel (Version 14.7258.5000).

#### Step 1: Fuzzification − Determination of fuzzy sets

The process of fuzzification implies that concrete (measured) values are assigned to fuzzy quantities. That means that the degree to which a certain situation fulfils certain properties is determined.

For the definition of the fuzzy sets the measured CCF_max_ values were used. Precisely, for each parameter ($\dot {\text{Q}}$, $\dot {\text{V}}$O_2musc_, $\dot {\text{V}}$O_2pulm_) box-plots were applied for the transient limits of the fuzzy sets. Overall, five limits for each physiologic parameter have been derived by using standard categorizations implying median and interquartile range (IQR).

**Fig. 4:**
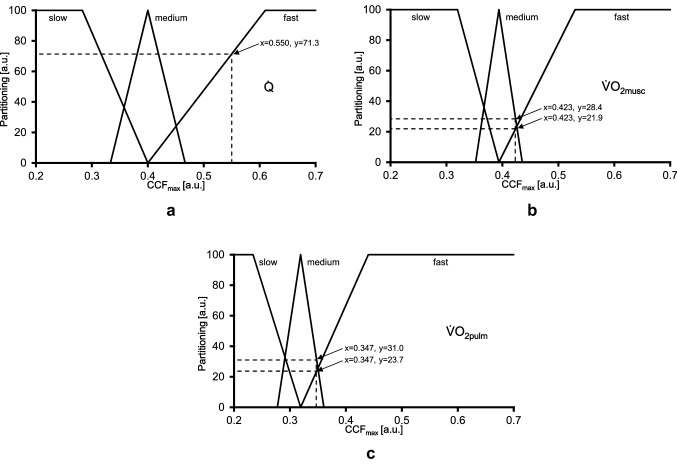
Derived fuzzy sets (solid lines) for cardiac output (${\dot{\text{Q}}}$, 4A), muscle ($\dot {\text{V}}$O_2musc_; 4B) and pulmonary oxygen uptake ($\dot {\text{V}}$O_2pulm_; 4C) kinetics. Transition values of the maxima of the cross-correlation function values (CCF_max_) on the ordinate are the limits illustrated in Table 2. For each parameter an example for the determination of the confidence values has been added (dotted lines)

The five limits of the fuzzy sets are shown for each parameter in Table 2 and illustrated in Fig. 4 (see below).

**Table 2:** Description of the derived fuzzy sets for cardiac output ($\dot {\text{Q}}$), muscle ($\dot {\text{V}}$O_2musc_), and pulmonary oxygen uptake ($\dot {\text{V}}$O_2pulm_) kinetics

Derived fuzzy sets based on CCF_max_ values		X values		
		$\dot {\text{Q}}$	$\dot {\text{V}}$O_2musc_	$\dot {\text{V}}$O_2pulm_
1	Median – 1.5 × IQR (last value within range)	0.283	0.320	0.234
2	Median – 0.5 × IQR	0.333	0.352	0.277
3	Median	0.400	0.394	0.319
4	Median + 0.5 × IQR	0.467	0.435	0.361
5	Median + 1.5 × IQR (last value within range)	0.610	0.530	0.440

IQR: interquartile range; CCF_max_: maxima of the cross-correlation function

#### Step 2: Inference − Determination of confidence values

The inference step implies that pre-defined calculation rules are applied to determine the degree of affiliation of a property. For each parameter and each participant the confidence values were calculated using the derived fuzzy sets (Combs method) introduced by Combs & Andrews (1998). The confidence values are in the range from 0 to 100 as illustrated in Fig. 4.

**Fig. 5:**
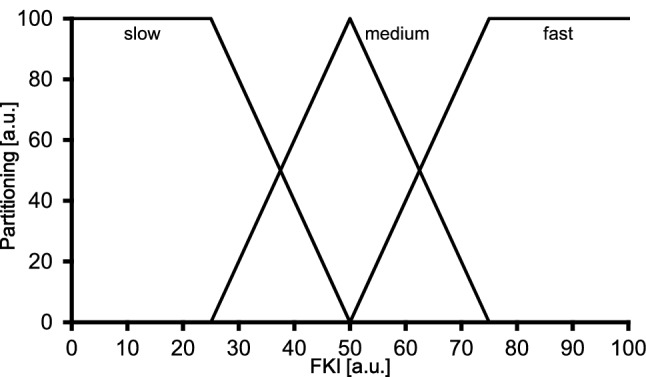
Visual representation of the defined graduations of the kinetic classifications, based on Table 3

In case of overlapping fuzzy set limits (see Fig. 4B and C), two confidence values can be determined. In such cases, the maximum (y-value) of the two values was used for further analysis (Fig. 5).

#### Step 2: Classification − Determination of regulation degrees

For a general categorization of the FKI, a balanced graduation has been defined, resulting in five kinetic classifications (see Table 3) with an overall range from 0 to 100.

**Table 3:** Definition of the graduation of the kinetic characteristics

Kinetic classification		FKI	
		Lower limit	Upper limit
1	Very slow	≥ 0	≤ 25
2	Slow	> 25	≤ 37.5
3	Medium	> 37.5	≤ 62.5
4	Fast	> 62.5	≤ 75
5	Very fast	> 75	≤ 100

FKI: Fuzzy Kinetics Index

For instance, a FKI value smaller than 25 represents very slow and greater than 75 very fast kinetics of the combined cardiorespiratory and pulmonary system.

In between (range: 25 to 75) the kinetic classification implies a linear transition from (very) slow to (very) fast kinetics for the FKI values.

#### Step 3: Defuzzification − FKI estimation

**Fig. 6:**
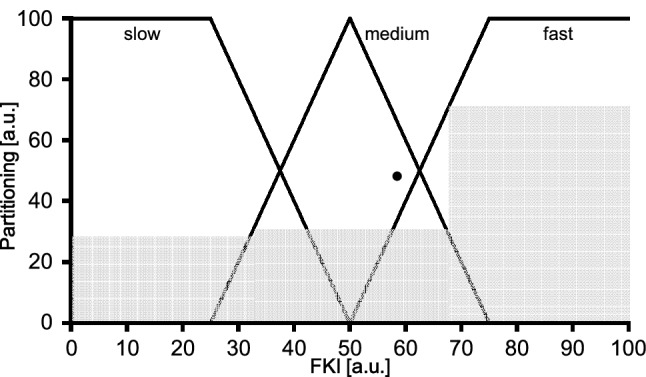
Example of a Fuzzy Kinetics Index (FKI) calculation as combined center of the rectangle areas (black dot) based on the three physiologic parameters cardiac output, pulmonary, and muscle oxygen uptake kinetics

The defuzzification is defined as the conversion of fuzzy quantities into concrete numerical values. For calculation of FKI comprising the kinetic responses of $\dot {\text{Q}}$, $\dot {\text{V}}$O_2pulm_ and $\dot {\text{V}}$O_2musc_, the average of maxima (AOM) method was applied (e.g., van Leekwijck & Kerre 1999). By means of AOM each kinetic classification (slow, medium, fast), based on the initial kinetic variables ($\dot {\text{Q}}$, $\dot {\text{V}}$O_2pulm_, $\dot {\text{V}}$O_2musc_) were simplified as rectangles, at which the start and end points (x-axis) are determined (see Fig. 6). Next, the average value of the start and end point was estimated.

Finally, the FKI value was calculated with the following equation as combined center of the rectangle areas of the three estimated kinetic regulation degrees (slow, medium, fast).

2 $$FKI=\frac{\sum_{i=1}^{3}\left({\stackrel{-}{x}}_{i}\bullet {max}_{i}\right)}{\sum_{i=1}^{3}\left({max}_{i}\right)}$$

where the index *i* denotes the kinetic regulation degree (1 = slow, 2 = medium, 3 = fast); $\stackrel{-}{x}$ is the average value of the start and end point, and ${max}_{i}$ is the maximum derived from the confidence values (see step 2 above).

The resulting FKI is then in the range from 0 to 100 (see Fig. 6). For a basic evaluation of the FKI the defined upper and lower boundaries from Table 3 have been applied.

### Statistical analysis

As pretest for subsequent statistical analysis, Kolmogorov–Smirnov test of normality with Lilliefors significance correction was applied for data of interest and especially for evaluation of the FKI frequency distribution. For correlation analyses either Pearson’s product-moment or Spearman's rank correlation coefficients was applied. P < 0.05 was set for statistical significance.

## Results

**Fig. 7:**
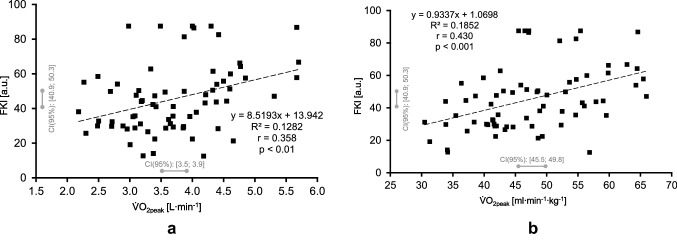
Relationship between the Fuzzy Kinetics Index (FKI) with absolute (7A) and relative (7B) peak oxygen uptake ($\dot {\text{V}}$O_2peak_); CI = confidence interval

For both absolute and relative $\dot {\text{V}}$O_2peak_ a significant correlation has been observed with FKI, whereas the correlation coefficient is higher for relative (r = 0.430, p < 0.001) compared to absolute (r = 0.358, p < 0.01) $\dot {\text{V}}$O_2peak_ (Fig. 7).

In this regard, correlations between $\dot {\text{V}}$O_2peak_ (absolute or relative) with either $\dot {\text{Q}}$, $\dot {\text{V}}$O_2pulm_ or $\dot {\text{V}}$O_2musc_ kinetics show smaller significant correlation coefficients than with FKI (see Table 4).

**Table 4:** Correlation analyses between Fuzzy Kinetics Index (FKI), absolute (abs) and relative (rel) peak oxygen uptake ($\dot {\text{V}}$O_2peak_), as well as kinetic responses (time constants) of perfusion ($\dot {\text{Q}}$), pulmonary ($\dot {\text{V}}$O_2pulm_), and muscle oxygen uptake ($\dot {\text{V}}$O_2musc_)

	$\dot {\text{V}}$O_2peak_ (abs)	$\dot {\text{V}}$O_2peak_ (rel)	$\dot {\text{Q}}$	$\dot {\text{V}}$O_2pulm_	$\dot {\text{V}}$O_2musc_
FKI	*r* = 0.358	*r* = 0.430	*r* = − 0.536	*r* = − 0.724	*r* = − 0.753
	*p* < 0.01	*p* < 0.001	*p* < 0.001	*p* < 0.001	*p* < 0.001
$\dot {\text{V}}$O_2peak_ (abs)		*r* = 0.773	*r* = − 0.323	*r* = − 0.217	*r* = − 0.325
		*p* < 0.001	*p* < 0.01	*p* > 0.05	*p* < 0.01
$\dot {\text{V}}$O_2peak_ (rel)			*r* = − 0.340	*r* = − 0.260	*r* = − 0.287
			*p* < 0.01	*p* < 0.05	*p* < 0.05
$\dot {\text{V}}$				*r* = 0.167	*r* = 0.377
				*p* > 0.05	*p* < 0.01
$\dot {\text{V}}$O_2pulm_					*r* = 0.746
					*p* < 0.001

No significant correlations have been found between FKI and age, height, body mass or body mass index (p > 0.05 each).

**Fig. 8:**
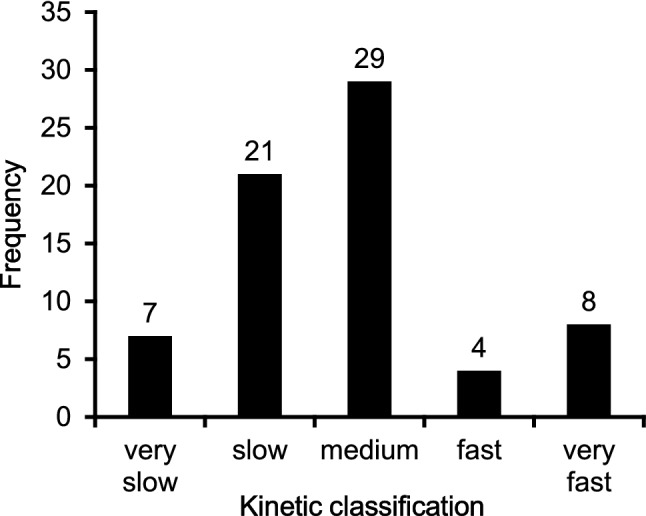
Frequency distribution of the Fuzzy Kinetics Indices (FKI) grouped by the defined classifications

The frequency distribution of the FKI values is in contrast to normal distribution (p < 0.001; Fig. 8).

## Discussion

The aim of the study was to establish a novel index using fuzzy logic procedures conflating cardiorespiratory and pulmonary kinetics during dynamic exercise as representative indicator for exercise tolerance and sport performance. Further, the FKI was applied to measured physiological data to establish correlations with $\dot {\text{V}}$O_2peak_ and additional parameters of interest.

The most significant results of the present study are the following:

- Significant moderate correlations have been observed between FKI and absolute (r = 0.358, p < 0.01) as well as relative (r = 0.430, p < 0.001) $\dot {\text{V}}$O_2peak_.
- No significant correlations could be estimated between FKI and age, height, body mass or body mass index (p > 0.05 each).
- The frequencies of the FKI classification showed significant contradictions to normal distribution (p < 0.001).

### Approach development and application

The results show that the main objective of the study was successfully reached; precisely for the FKI, significant correlations have been estimated with $\dot {\text{V}}$O_2peak_. This connection was previously assumed due to the physiologic link between the cardiorespiratory and pulmonary system and their capacitive and regulatory characteristics. These properties are fundamentally based on the significant correlations between $\dot {\text{V}}$O_2_ kinetics with $\dot {\text{V}}$O_2peak_ (Chillibeck et al. 1996; Burnley & Jones 2007). Further, it seems that additional variables such as age, height, body mass, or body mass index have no significant impact on FKI.

### Limitations

The CCF_max_ values are not a 100% representative proxy of the underlying dynamic physiologic responses. This was demonstrated for instance by Drescher (2018). Therefore, the CCF_max_ values are a practical approximation of the physiologic system as a response to dynamic exercise and represent fuzzy kinetic information.

The determinations of the fuzzy sets have been derived from data of 69 healthy participants. This is a clear limitation if FKI will be applied to other populations like children, diseased people or frail elderly due to possibly divergent fuzzy sets. Therefore, population-based fuzzy sets should be established for a more sophisticated application of the FKI.

An evaluation of absolute performance of the cardiorespiratory and pulmonary system is not possible with FKI. This is due to that FKI displays the relative changes – in the current case – during moderate WR intensity changes, e.g., below gas exchange threshold (GET). Moreover, above GET fuzzy sets have to be calculated to transfer the results of the current study from moderate into submaximal intensity ranges.

### Conclusion and future directions

It was demonstrated that the successful establishment of FKI for healthy participants in the moderate exercise intensity range can be applied as an indicator for sport performance and exercise tolerance. In this regard, FKI can be of interest for conditions where $\dot {\text{V}}$O_2max_ or $\dot {\text{V}}$O_2peak_ values are not available or cannot be measured due to motivational lacks of the participants or critical reasons (e.g., patients with arterial hypertension).

The significant correlations between FKI and $\dot {\text{V}}$O_2peak_ represent a physiological connection between the regulatory and the capacitive system and its exercise performance.

Further, a transfer of the methodology presented here is also possible to other physiological variables, parameters, and appropriate exercise protocols: specifically for τ of phase II $\dot {\text{V}}$O_2pulm_ kinetics. This appears to be particularly worthwhile if other variables are added to the holistic approach to estimating exercise performance.

Finally, FKI may be of interest, for instance, for patients with heart failure, chronic obstructive pulmonary disease, or other frail populations for an estimation of exercise tolerance without strenuous exhaustion.

## Supplementary Information

Below is the link to the electronic supplementary material.

Supplementary file1 (PDF 562 KB)

## Abbreviations

ACF [a.u.]: Auto-correlation function
AOM: Average of maxima
CCF [a.u.]: Cross-correlation function
CCF_max_[a.u.]: Maximum (peak) of cross-correlation function
FKI: Fuzzy kinetics index
GET: Gas exchange threshold
HR [min^−^^1^]: Heart rate
IQR: Interquartile range
PRBS: Pseudo random binary sequence
$\dot {\text{Q}}$ [L·min^−^^1^]: Cardiac output / perfusion
SV [ml]: Stroke volume
τ [s]: Time constant
$\dot {\text{V}}$O_2_ [L·min^−^^1^]: Oxygen uptake
$\dot {\text{V}}$O_2max_ [L·min^−^^1^]: Maximal oxygen uptake
$\dot {\text{V}}$O_2musc_ [L·min^−^^1^]: Exercising muscle oxygen uptake
$\dot {\text{V}}$O_2peak_ [L·min^−^^1^]: Peak oxygen uptake
$\dot {\text{V}}$O_2pulm_ [L·min^−^^1^]: Pulmonary oxygen uptake
WR [W]: Work rate
