# Correction to: The Fuzzy Kinetics Index: an indicator conflating cardiorespiratory kinetics during dynamic exercise

**DOI:** 10.1007/s00421-021-04665-w

**Published:** 2021-04-07

**Authors:** U. Drescher

**Affiliations:** German Sport University Cologne, Am Sportpark Müngersdorf 6, 50933 Cologne, Germany

## Correction to: European Journal of Applied Physiology 10.1007/s00421-021-04611-w

The original version of this article unfortunately contained a mistake. There is one small mistake in Table 4.

The corrected Table 4 is given in the following page.

**Table 4:** Correlation analyses between Fuzzy Kinetics Index (FKI), absolute (abs) and relative (rel) peak oxygen uptake ($\dot {\text{V}}$O_2peak_), as well as kinetic responses (time constants) of perfusion ($\dot {\text{Q}}$), pulmonary ($\dot {\text{V}}$O_2pulm_), and muscle oxygen uptake ($\dot {\text{V}}$O_2musc_)

	$\dot {\text{V}}$O_2peak_ (abs)	$\dot {\text{V}}$O_2peak_ (rel)	$\dot {\text{Q}}$	$\dot {\text{V}}$O_2pulm_	$\dot {\text{V}}$O_2musc_
FKI	*r* = 0.358	*r* = 0.430	*r* = − 0.536	*r* = − 0.724	*r* = − 0.753
	*p* < 0.01	*p* < 0.001	*p* < 0.001	*p* < 0.001	*p* < 0.001
$\dot {\text{V}}$O_2peak_ (abs)		*r* = 0.773	*r* = − 0.323	*r* = − 0.217	*r* = − 0.325
		*p* < 0.001	*p* < 0.01	*p* > 0.05	*p* < 0.01
$\dot {\text{V}}$O_2peak_ (rel)			*r* = − 0.340	*r* = − 0.260	*r* = − 0.287
			*p* < 0.01	*p* < 0.05	*p* < 0.05
$\dot {\text{Q}}$				*r* = 0.167	*r* = 0.377
				*p* > 0.05	*p* < 0.01
$\dot {\text{V}}$O_2pulm_					*r* = 0.746
					*p* < 0.001

