# Supplementary material for: The Fuzzy Kinetics Index: an indicator conflating cardiorespiratory kinetics during dynamic exercise
Source: Eur J Appl Physiol. 2021 Feb 18;121(5):1349–57. doi: 10.1007/s00421-021-04611-w (PMC8064983; doi:10.1007/s00421-021-04611-w)
Supplement: Supplementary file 1 — Supplementary file1 (PDF 562 KB) [file 421_2021_4611_MOESM1_ESM.pdf]

FKI - Fuzzy Kinetics Index: Supplementary material

Q' – cardiac output / perfusion

SLOW

=**IF** ((CCF_{max}>0) AND (CCF_{max} ≤0.283))

THEN ("100")

ELSE ("")

=**IF** ((CCF_{max}>0.283) AND (CCF_{max} ≤0.400))

THEN (-854.7*CCF_{max} +341.88)

ELSE ("")

FAST

=**IF** ((CCF_{max} >0.400) AND (CCF_{max} ≤0.610))

THEN (476.19* CCF_{max} -190.48)

ELSE ("")

=**IF** ((CCF_{max} >0.610) AND (CCF_{max} ≤1.00))

THEN ("100")

ELSE ("")

MEDIUM

=**IF** ((CCF_{max} >0.333) AND (CCF_{max} ≤0.400))

THEN (1497.2* CCF_{max} -498.86)

ELSE ("")

=**IF** ((CCF_{max} >0.400) AND (CCF_{max} ≤0.467))

THEN (-1497.2* CCF_{max} +698.86)

ELSE ("")

$V'O_{2pulm}$ – pulmonary oxygen uptake

SLOW

=**IF** ((CCF_{max} >0) AND (CCF_{max} ≤0.234))

THEN ("100")

ELSE ("")

=**IF** ((CCF_{max} >0.234) AND (CCF_{max} ≤0.319))

THEN (-1176.3* CCF_{max} +375.25)

ELSE ("")

FAST

=**IF** ((CCF_{max} >0.319) AND (CCF_{max} ≤0.440))

THEN (826.53* CCF_{max} -263.67)

ELSE ("")

=**IF** ((CCF_{max} >0.440) AND (CCF_{max} ≤1.000))

THEN ("100")

ELSE ("")

MEDIUM

=**IF** ((CCF_{max} >0.277) AND (CCF_{max} ≤0.319))

THEN (2406.1* CCF_{max} -667.58)

ELSE ("")

=**IF** ((CCF_{max} >0.319)AND (CCF_{max} ≤0.361))

THEN (-2406.1* CCF_{max} +867.58)

ELSE ("")

***$V'O_{2musc}$* – muscle oxygen uptake**

SLOW

=**IF** ((CCF_{max} >0.000) AND (CCF_{max} ≤0.320))

THEN ("100")

ELSE ("")

=**IF** ((CCF_{max} >0.320) AND (CCF_{max} ≤0.394))

THEN (-1352.1* CCF_{max} +532.41)

ELSE ("")

FAST

=**IF** ((CCF_{max} >0.394) AND CCF_{max} ≤0.530))

THEN (733.99* CCF_{max} -289.01)

ELSE ("")

=**IF** ((CCF_{max} >0.530) AND (CCF_{max} ≤1.000))

THEN ("100")

ELSE ("")

MEDIUM

=**IF** ((CCF_{max} >0.352) AND (CCF_{max} ≤0.394))

THEN (2398.2* CCF_{max} -844.3)

ELSE ("")

=**IF** ((CCF_{max} >0.394) AND (CCF_{max} ≤0.435))

THEN (-2398.2* CCF_{max} +1044.3)

ELSE ("")
